# The effects of autophagy on the replication of Nelson Bay orthoreovirus

**DOI:** 10.1186/s12985-019-1196-7

**Published:** 2019-07-18

**Authors:** Xiao-Li Tao, Wei Zhao, Wei Tong, Xiao-Fang Wang, Li-Li Dou, Jiang-Man Chen, Nian Liu, Ying Lu, Yi-Bo Zhang, Xu-Peng Jin, Yan-Fei Shen, Hong-Yan Zhao, Hong Jin, Yong-Gang Li

**Affiliations:** 1 Department of Pathogenic Microbiology, College of Basic Medical Sciences, China Medical University, No. 77, Puhe Road, Shenyang North New Area, Shenyang City, 110013 Liaoning Province People’s Republic of China; 2 Department of Pathogenic Microbiology, College of Basic Medical Sciences, Jinzhou Medical University, No. 40, the Third Section of SongPo Rd, Jinzhou City, 121200 Liaoning Province China

**Keywords:** Autophagy, Nelson Bay, Orthoreovirus replication, μNS

## Abstract

**Background:**

Nelson Bay orthoreovirus (NBV) was first isolated over 40 years ago from a fruit bat in Australia. Normally, NBV does not cause human diseases, but recently several NBV strains have been associated with human respiratory tract infections, thus attracting clinical attention. Autophagy, an evolutionarily conserved process in eukaryotic cells, degrades intracellular substrates, participates in multiple physiological processes, and maintains cellular homeostasis. In addition, autophagy is intimately involved in viral infection.

**Methods:**

A new strain of NBV, isolated from a patient with a respiratory tract infection who returned to Japan from Bali, Indonesia, in 2007, was used in this study. NBV was rescued using a reverse genetics system involving cotransfection of BHK cells with 11 plasmids (pT7-L1 MB, pT7-L2 MB, pT7-L3 MB, pT7-M1 MB, pT7-M2 MB, pT7-M3 MB, pT7-S1 MB, pT7-S2 MB, pT7-S3 MB, pT7-S4 MB, and pcDNA3.1-T7), yielding NBV-MB. Recovered viruses were confirmed by immunofluorescence. The effect of NBV-MB on autophagy was evaluated by measuring the LC3-I/II proteins by immunoblot analysis after infection of BHK cells. Furthermore, after treatment with rapamycin (RAPA), 3-methyladenine (3-MA), chloroquine (CQ), or plasmid (GFP-LC3) transfection, the changes in expression of the LC3 gene and the amount of LC3-I/II protein were examined. In addition, variations in viral titer were assayed after treatment of BHK cells with drugs or after transfection with plasmids pCAGM3 and pCAGS3, which encode virus nonstructural proteins μNS and σNS, respectively.

**Results:**

NBV-MB infection induced autophagy in host cells; however, the level of induction was dependent on viral replication. Induction of autophagy increased viral replication. By contrast, inhibiting autophagy suppressed NBV replication, albeit not significantly. The NBV-MB nonstructural protein μNS was involved in the induction of autophagy with viral infection.

**Conclusions:**

NBV-MB infection triggered autophagy. Also, the NBV nonstructural protein μNS may contribute to augmentation of autophagy upon viral infection.

## Background

Orthoreoviruses are double-stranded nonenveloped RNA viruses belonging to family Reoviridae. Their genomes consist of ten dsRNA segments, which are classified on the basis of size as large (L1–L3), medium (M1–M3), and small (S1–S4). Based on the ability to induce cell-to-cell fusion in infected cells, orthoreoviruses are divided into fusogenic and nonfusogenic subgroups [1]. Avian orthoreovirus (ARV), baboon orthoreovirus (BRV), reptilian orthoreovirus, Broome reovirus (BroV), and Nelson Bay orthoreovirus (NBV) belong to the fusogenic subgroup, whereas mammalian orthoreovirus (MRV) is in the nonfusogenic subgroup [1–3].

Nonfusogenic MRVs are very common and normally cause asymptomatic infections in humans. Fusogenic orthoreoviruses can cause severe diseases in infected animals, but ercf ordinarily not pathogenic in humans. However, a new fusogenic orthoreovirus named Melaka (Mel) virus was isolated in Malaysia in 2006. Mel virus causes acute respiratory tract infection in humans [4]. The genome of this virus was related to strains Nelson Bay (NB) and Pulau, which were isolated from bats in Australia and Malaysia, respectively [5]. Subsequently, other strains have been isolated from patients suffering from severe respiratory illness in Malaysia and Hong Kong [6–9]. These strains have now been isolated from humans with acute respiratory tract infections, implying that the virus is a zoonotic pathogen. In this study, a new strain isolated from a patient with respiratory tract infection who returned to Japan from Bali, Indonesia, in 2007 was used. This strain was named Miyazaki-Bali/2007 (MB) virus [10, 11].

Autophagy is an evolutionarily conserved process in eukaryotic cells that degrades intracellular substrates, participates in multiple physiological processes, and maintains cellular homeostasis [12]. During autophagy, cytoplasmic components, including bulk cytoplasm and organelles, are sequestered into double-membrane vesicles termed autophagosomes and delivered to the lysosome for degradation [13]. The double-membrane autophagosomes are a hallmark of autophagy. During formation of the autophagosome, LC3 (microtubule-associated protein-1 light chain 3) is converted to lipidated LC3-II, which accumulates in the autophagic membrane. The LC3-II/β-actin ratio is related to the number of autophagosomes [12, 14]. p62 (SQSTM1), another marker of autophagy that is degraded by the autophagy–lysosome pathway, interacts with LC3 directly [15]. These proteins are often used to evaluate and distinguish between different types of autophagy. The autophagy pathway and/or the autophagy machinery plays a critical role in the cell’s antiviral defenses, and this pathway restricts the virulence of some viruses in organisms ranging from plants to mammals [16, 17]. However, some viruses are apparently unaffected by autophagy, and many viruses have evolved to escape or exploit this mechanism to promote their survival and replication in various ways [18–21].

Many viruses successfully evade autophagy and even exploit the autophagic machinery to promote viral replication [22]. Viruses modulate the behavior of host cells in several ways to obtain replicative advantages, and one such strategy involves modulation of host cell autophagy. Many studies recently reported that viral infections affect or enlist the autophagic process in host cells [23]. Interactions between viruses and the host autophagic machinery are complex and virus-specific: Viruses can utilize autophagy as a platform for replication, while autophagy can favor or limit viral replication, thereby potentially contributing to the pathogenesis of specific viruses.

The ARV strain induces autophagy in both primary chicken embryonic fibroblasts (CEFs) and mammalian Vero cells. Viral infection increases the number of double-membrane vesicles, detected as puncta of transiently expressed GFP-LC3 and elevated levels of the autophagy marker LC3-II [24]. ARV infection triggers autophagy through the PI3K/Akt/mTOR pathway [24]. Specifically, the nonstructural protein p17 of ARV functions as an activator of autophagy [25]. Similarly, the Muscovy duck reovirus nonstructural protein σNS triggers autophagy and promotes virus replication [26]. Thus, nonstructural proteins of reovirus may influence induction of autophagy by viral infection. Although the relationship between autophagy and MRV replication has not been shown directly, as in the case of ARV, MRV replicates more rapidly in cells with an intact protein kinase R (PKR) pathway, suggesting that autophagy promotes MRV replication [18, 24, 27]. In addition to ARV and MRV, several other viruses induce autophagy, which in turn plays important roles in their life cycles and pathogenesis. Human tumor viruses (γ-herpesviruses), Epstein-Barr virus (EBV), Kaposi’s sarcoma-associated human herpes virus type 8 (KSHV), hepatitis C virus (HCV), rotavirus, and hepatitis B virus (HBV) have evolved various strategies to either subvert or activate the autophagic pathway to promote their own replication and pathogenesis [28, 29]. To date, the role of autophagy in infection and pathogenesis of NBV has not been investigated. Rapamycin (RAPA) is autophagy inducer, whereas 3-methyladenine (3-MA) and chloroquine (CQ) is autophagy inhibitors. In this study, we examined the NBV-MB to determine whether NBV-MB infection affects autophagy. To the best of our knowledge, this is the first report to describe how NBV-MB infection alters and enlists autophagy within the host cell.

Our findings revealed that NBV-MB infection indeed induced autophagy in host cells, and that induction of autophagy was dependent on viral replication. Activation of autophagy increased viral replication. By contrast, inhibition of autophagy did not decrease replication. The μNS protein of NBV-MB was involved in augmentation of autophagy during viral infection. Our findings provide the first evidence that NBV-MB affects the process of autophagy to promote its own replication, and thus highlight the role of autophagy in NBV-MB pathogenesis.

## Materials and methods

### Cells and viruses

L929, BHK, and human embryonic kidney 293 T cells were obtained from the American Type Culture Collection (Manassas, VA, USA). All cells were grown in Dulbecco’s modified Eagle’s medium (DMEM; Gibco) supplemented with 5% fetal bovine serum (FBS; Gibco), 100 U/ml penicillin, and 100 μg/ml streptomycin (Invitrogen). The original NBV-MB strain was isolated from a patient with acute respiratory tract infection in Japan in 2007 [10], and the NBV used in this study was recovered from patient samples using a reverse genetics system [30]; the resulting virus was named NBV-MB. Viruses were propagated in L929 cells and titers were determined using plaque assays in L929 cell monolayers [31].

### Plasmids, antibodies, and reagents

Viral RNA was extracted from infected cells and subjected to RT-PCR to amplify the genes M3 and S3. The M3 and S3 amplified segments were cloned into vector pCAG to yield pCAGM3 and pCAGS3, respectively. Cloned plasmids were confirmed by sequencing. The human GFP-LC3 plasmid was kindly provided by Dr. Y.B. Zhang [32]. A polyclonal rabbit anti-LC3-I/II antibody (ab128025, Cambridge, UK) was purchased from Abcam, and a mouse monoclonal antibody against β-actin (TA-09) was purchased from Zhongshan Golden Bridge (Beijing, China, TA-09). 3-MA (3-methyladenine, HY-19312) and CQ (chloroquine, HY-17589) were from MedChemExpress (Monmouth Junction, NJ, USA), and RAPA (rapamycin, S1824) was from Beyotime (Haimen, China). The anti-μNS rabbit serum was prepared in-house. Briefly, the M3 segment of the NBV genome encoding μNS was cloned into a bacterial expression vector and the recombinant plasmid was transformed into *Escherichia coli* BL21 (DE3) cells. The NBV M3 fusion protein was purified on Ni-NTA agarose and utilized as an antigen to immunize rabbits and obtain an anti-NBV μNS polyclonal antibody.

### Rescue of NBV using a reverse genetics system

Monolayers of BHK cells (8 × 10^5^ cells) seeded on 6-well plates (Corning) were cotransfected with 11 plasmids. Ten of these plasmids contained cloned cDNAs corresponding to each of the 10 full-length RNA gene segments of NBV-MB (L1–L3, M1–M3, and S1–S4); the eleventh, pcDNA3.1-T7RNAPOl, encodes the T7 polymerase protein. pcDNA3.1-T7RNAPOl was kindly provided by Professor Chunlai Jiang (School of Life Science, Jilin Medical University). Two microliters of TransIT-LT1 transfection reagent (Mirus) was used per microgram of plasmid DNA. The amount of each plasmid used for transfection was as follows: pT7-L1 MB, 0.66 μg; pT7-L2 MB, 0.66 μg; pT7-L3 MB, 0.66 μg; pT7-M1 MB, 0.58 μg; pT7-M2 MB, 0.58 μg; pT7-M3 MB, 0.58 μg; pT7-S1 MB, 0.5 μg; pT7-S2 MB, 0.5 μg; pT7-S3 MB, 0.5 μg; pT7-S4 MB, 0.5 μg; and pcDNA3.1-T7, 0.5 μg. After 6 days of incubation, recombinant virus from transfected cells was amplified through L929 infection, and confirmed by cytopathic effect (CPE) and immunofluorescence assay (IFA).

### Immunofluorescence assay

Monolayers of BHK cells were seeded onto 12-well plates and infected with viruses generated using the reverse genetics system. After incubation for 24 h, cells were fixed with PBS containing 4% paraformaldehyde, washed with PBS, and incubated with μNS antiserum prepared in-house (unpublished data). After three washes with PBS, cells were incubated with Alexa Fluor 594-conjugated goat anti-rabbit IgG secondary antibody (Invitrogen) diluted 1:500 in PBS. Images were acquired under a fluorescent microscope (LEICA, DMI3000B). Images of integrated density (IntDen) within cells obtained under the confocal fluorescence microscope were analyzed using software Image J. The results of data analysis were presented as a histogram.

### Immunoblot analysis

Since the lipidated form of LC3-II, a hallmark of autophagy induction, is widely used to evaluate autophagic activity [33], expression of LC3-II was measured by immunoblotting to monitor autophagy after viral infection. BHK cells in 60 mm dishes were infected with NBV-MB at a multiplicity of infection (MOI) of 1 per cell. One hour after infection, the medium was removed and replaced with DMEM supplemented with 2% FBS for the duration of the experiment. Virus-infected cells were harvested at the designated time points. The cells were lysed in buffer consisting of 25 mM Tris–HCl pH 7.4, 150 mM NaCl, 1% NP-40, 1% sodium deoxycholate, and 0.1% SDS. After centrifugation, soluble protein fractions were size-fractionated in a 12% sodium dodecyl sulfate (SDS)-polyacrylamide gel electrophoresis (PAGE) gel and transferred to nitrocellulose membranes (Millipore) using a Trans-Blot cell (Bio-Rad Laboratories, Inc. California, USA). The membranes were blocked for 2 h at room temperature with 5% (m/v) skim milk in Tris buffer, 0.05% (v/v) Tween 20, pH 7.4 (TBST), washed three times in TBST, and then incubated with a rabbit anti-LC3-I/II antibody (diluted 1:1000 in TBST) and a mouse anti-actin antibody (Zhongshan Golden Bridge, Beijing, China) (diluted 1:3000 in TBST). Finally, membranes were incubated with HRP-conjugated anti-mouse or anti-rabbit IgG secondary antibodies (Sigma, Shanghai, China) diluted 1:3000 in TBST. Expression of LC3-I/II and β-actin was detected using Chemi-Lumi One Ultra (Bio-Rad). All experiments were performed three times. Integrated density (IntDen) images were analyzed using Image J. Differences in the level of autophagy were evaluated by calculating the LC3-II/β-actin ratio.

### Virus infection and drug treatments

To determine whether the NBV-MB strain induces autophagy, 1 × 10^6^ BHK cells were seeded in 60 mm cell culture dishes, incubated overnight, and then infected with NBV at a MOI of 1. After 1 h adsorption, the supernatants were removed, and the cells were washed three times with PBS; subsequently, the cells were cultured in DMEM supplemented with 2% FBS. Infected cells treated with 100 nM RAPA, 20 μM CQ, or 10 mM 3-MA (all diluted in PBS), as well as cells infected with inactivated viruses and mock-infected cells, were collected for analysis of autophagy induction. For these experiments, cells were infected at a MOI of 1, followed by treatment with drugs for 2 h. Specifically, BHK cells were treated with 10 mM 3-MA or 100 nM RAPA for 2 h and then infected with NBV-MB at a MOI of 1. For the CQ treatment group, viruses were diluted in 20 μM CQ. PBS treatment was used as a control. After treatment, BHK cells were infected with NBV at a MOI of 1. Cellular RNA was extracted at the designated time points and subjected to real-time PCR to determine the amount of *LC3* mRNA. Heat inactivation of NBV-MB was performed by heating for 30 min at 70 °C. Similar treatments were performed on BHK cells. Samples exposed to each treatment were collected and subjected to western blotting to detect autophagy-related proteins. Supernatant from the cells treated with 100 nM RAPA, 10 mM 3-MA, and 20 μM CQ was collected and examined in plaque assays to determine viral titers. Plaque assays were performed on L929 cell monolayers as previously described [31].

### Real-time PCR assay

Cellular RNA was extracted using Trizol (Invitrogen, Shanghai, China), and cDNA was synthesized using Prime Script™ RT Master Mix (TaKaRa, Dalian, China). qPCR was performed with SYBR Premix Dimer Eraser™ (TaKaRa, Dalian, China) on an Applied Biosystems 7500 fast real-time PCR cycler (Applied Biosystems, Foster City, CA, USA). Primers used for qRT-PCR were as follows: *LC3* F, GGAGACATTCGGGACAGCAA; *LC3* R, GTCCGCTGGTAACATCCCTT; *β-actin* F, GCACCACACCTTCTACAATGAG; *β-actin* R, CAGAGGCATACAGGGACAGC. qPCR was performed with an initial incubation of 30 s at 95 °C, followed by amplification for 40 cycles (95 °C for 5 s, 58 °C for 30 s, and 72 °C for 30 s). Each sample was run in triplicate. The specificity of amplification was confirmed by melting curve analysis. Expression of the *LC3* gene was calculated using the 2^-ΔΔCt^ method and normalized relative to *β-actin*.

### Statistical analysis

All data are expressed as the mean ± standard deviation (SD). When comparing three or more groups, statistical analysis was performed using one-way ANOVA (SPSS 13.0. software; SPSS Inc., Chicago, IL, USA), followed by Dunnett’s multiple range test. For all graphs, two asterisks indicate *p* < 0.01 and one asterisk indicates *p* < 0.05 (as determined by the Mann Whitney test for comparing two groups).

## Results

### Recovery of NBV-MB using a reverse genetics system

**Fig. 1:**
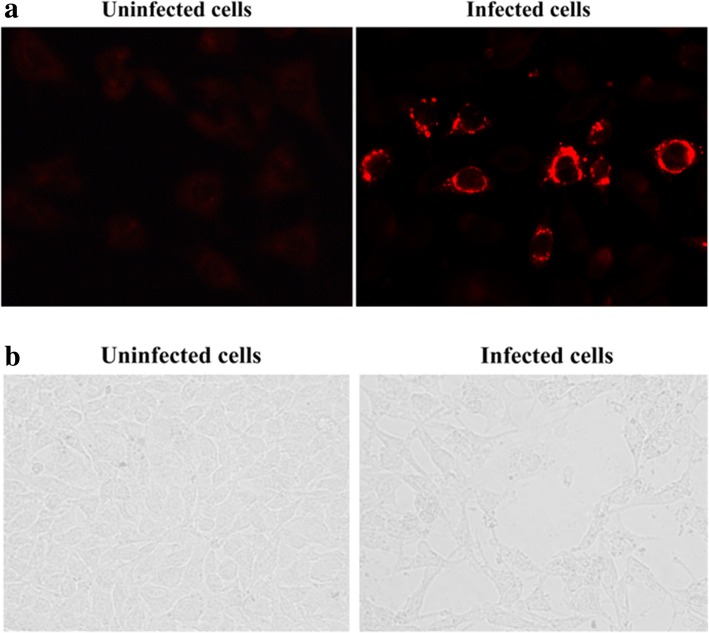
Production of NBV-MB by reverse genetics, confirmed by IFA and CPE. **a** BHK cells were transfected with viral plasmids and pcDNA3.1-T7. After 7 days, the viruses were collected and used to infect BHK cells, which were subjected to IFA after 24 h. **b** Viral infection induced CPE in BHK cells. BHK cells were infected with NBV-MB viruses recovered from the reverse genetics system; after 48 h, CPE was observed under a microscope

The viruses NBV-MB recovered from transfected BHK cells were used to infect BHK cells. After 24 h, the cells were fixed and subjected to IFA using antiserum against μNS of NBV-MB, prepared in rabbits (unpublished data). CPE was also observed under the microscope and compared with the negative control. In IFA, fluorescence signal was observed in infected cells, but not in mock-infected cells (Fig. 1a). Also, CPE was much higher in cells infected with recovered virus than in cells subjected to negative control infections (Fig. 1b). These results confirmed that NBV-MB was successfully recovered and could be used for subsequent experiments.

### Autophagy is induced by NBV-MB infection in BHK cells

**Fig. 2:**
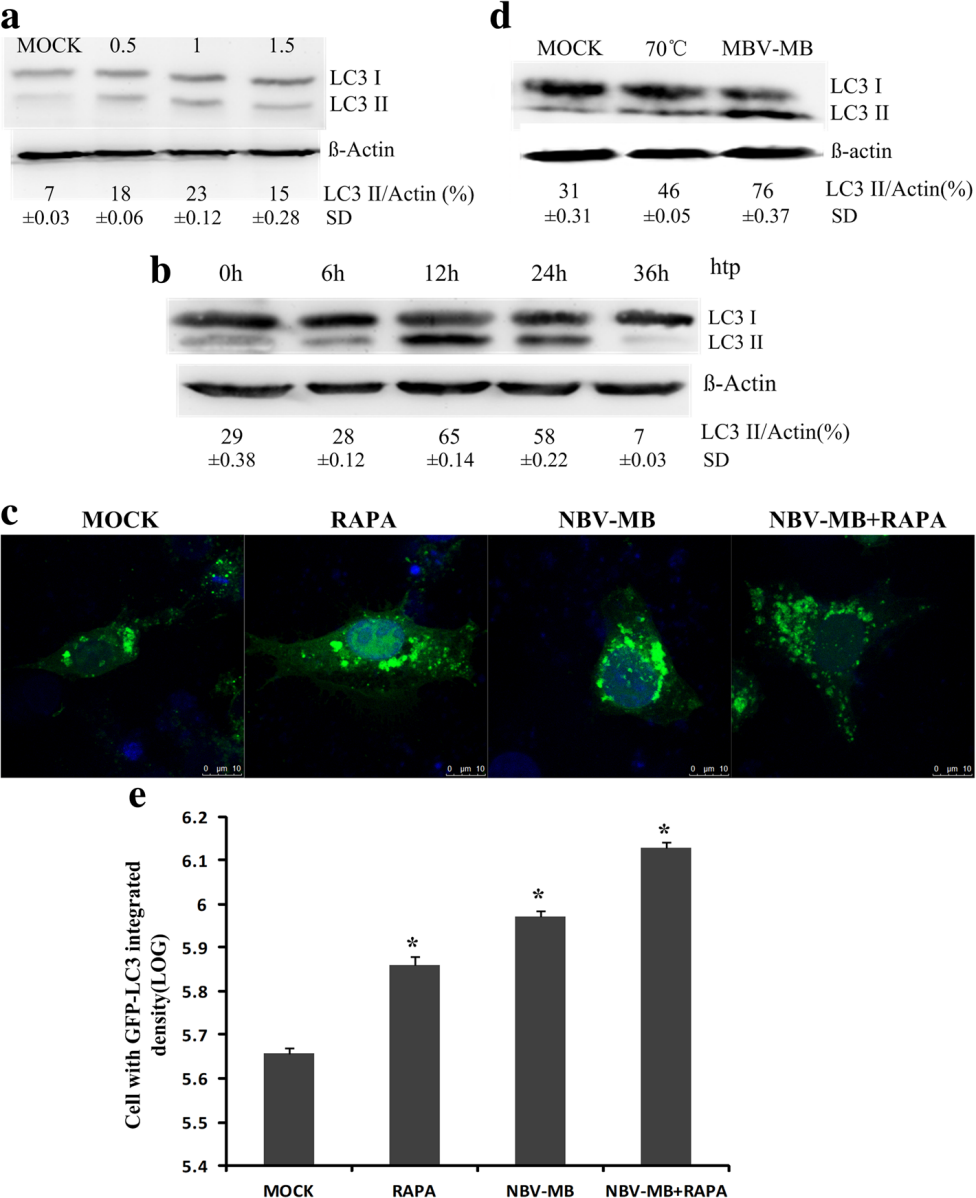
NBV-MB infection and autophagy in BHK cells. **a** BHK cells were cultured in 6-well plates and infected with the NBV-MB strain of NBV at a MOI of 0.5, 1, or 1.5. After 24 h, samples were collected and subjected to western blotting to detect LC3-I/II and β-actin. **b** BHK cells were cultured in 6-well plates and infected with NBV-MB at a MOI of 1. Samples were collected at 6, 12, 24, and 36 h post-infection and subjected to western blotting to detect LC3-I/II and β-actin. **c** BHK cells were transfected with the GFP-LC3 plasmid after 24 h and treated with RAPA (or not) for 2 h, followed by NBV-MB infection. **e** Integrated density of GFP-LC3 in cells displayed as a histogram. **d** BHK cells were cultured in 6-well plates and infected with the NBV-MB strain inactivated by heat treatment (70 °C for 30 min). After 24 h, the LC3 protein in cell lysates was measured by western blotting

BHK cells were infected with NBV-MB at a MOI of 0.5, 1, or 1.5. After 24 h, cells were lysed and immunoblotting was performed to detect LC3-II. Compared with mock infection, infection increased the expression of LC3-II, indicating that infection induced autophagy. As shown in Fig. 2a, conversion of LC3-I to LC3-II was significantly higher in NBV-MB-infected cells than in mock-infected cells. In addition, we infected BHK cells with NBV-MB at a MOI of 1, and then collected samples for immunoblotting after 0, 6, 12, 24, and 36 h. LC3-II expression was elevated after 12 and 24 h of virus infection, but decreased at 36 h (Fig. 2b).

To confirm these results, we monitored formation of GFP-LC3 dots. For this purpose, BHK cells were transfected with pEGFP-LC3 for 24 h and then infected or mock-infected with virus. After 24 h, the numbers of GFP-LC3 puncta were compared between infected and mock-infected cells. Because the puncta could not be counted clearly, we estimated the number of GFP-LC3+ cells using integrated density. As shown in Fig. 2c and e, GFP-LC3 dots induced by RAPA were used as a positive control. Integrated density of GFP-LC3 in cells was displayed as a histogram. The integrated density of GFP-LC3 in RAPA-treated and infected cells was higher than that in untreated and uninfected controls (*p < 0.05).

Given that infection triggered autophagy, we reasoned that autophagy may be necessary for productive viral infection. To explore this possibility, we infected BHK cells with normal virus or virus inactivated by heat treatment (70 °C for 30 min). We confirmed that heat-treated viruses could not produce CPE. Mock infection was used as a negative control. Viral infection was carried out at a MOI of 1, and cell lysates used for immunoblotting were collected after 24 h of infection. Normal virus, but neither mock infection nor inactivated virus infection, increased expression of LC3-II (Fig. 2d), indicating that autophagy induction depended on the productive viral infection. Together, these results show that autophagy is induced and enlisted for viral replication during NBV infection.

### Regulators of autophagy affect NBV-MB replication

**Fig. 3:**
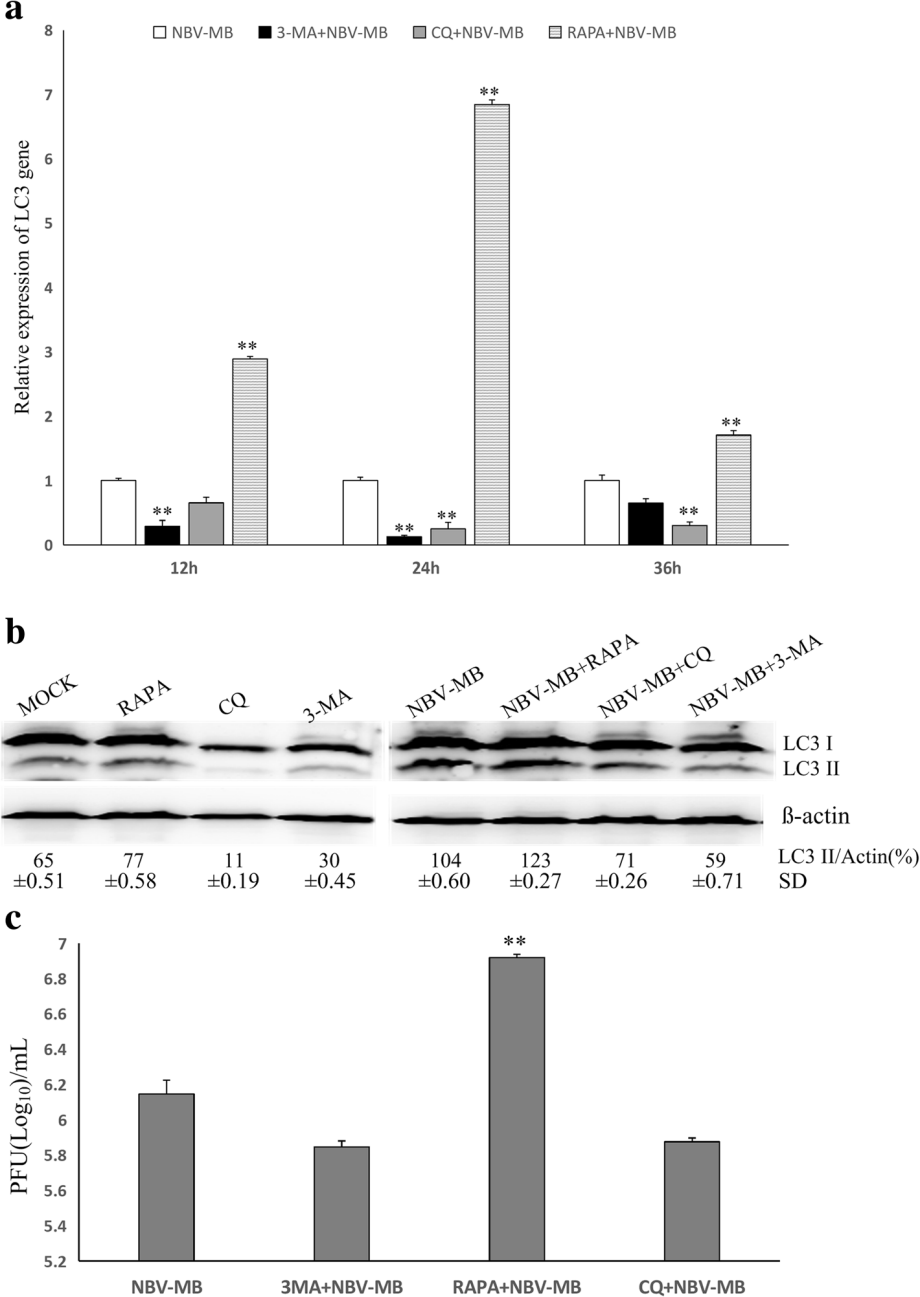
Effects of autophagy regulators on NBV-MB infection in BHK cells. BHK cells were treated with 3-MA (10 mM) and RAPA (100 nM) for 2 h and then infected with NBV-MB at a MOI of 1. After 12, 24, or 36 h, cellular RNA was extracted and subjected to real-time PCR to measure the level of *LC3* mRNA **a** After 24 h of treatment, LC3 proteins in cell lysates were measured by western blotting **b** After 24 h of infection, the viral titer in supernatants from samples exposed to each treatment was measured in a plaque assay (**c**)

The results described above showed that NBV-MB infection induced autophagy, but it remained unclear how autophagy affected NBV-MB replication. To address this, we treated cells with activators or inhibitors of autophagy and studied the effects on viral infection. The results revealed that RAPA increased the amount of *LC3* mRNA over time, reaching a peak after 24 h. By contrast, 3-MA and CQ decreased the level of *LC3* mRNA, which reached a minimum after 24 h (Fig. 3a).

Next, we investigated the effects of autophagy regulators on NBV-MB. For these experiments, BHK cells were treated with 3-MA and RAPA 2 h, and then infected with NBV at a MOI of 1. Cell lysates were also subjected to western blotting to assess expression of LC3-II protein. Compared with the PBS treatment, 3-MA and CQ decreased the LC3-II level, whereas RAPA increased it (Fig. 3b), in accordance with the effects of each drug on the mRNA level. Moreover, 24 h treatment with the autophagy inducer RAPA increased viral titer relative to infection alone (Fig. 3c). By contrast, the autophagy inhibitors 3-MA and CQ decreased viral titer, but the difference was not significant relative to infection alone.

Given that NBV-MB could induce autophagy, we speculated that viral proteins are involved in this phenomenon. Hence, we next investigated whether the nonstructural proteins σNS and μNS contributed to induction of autophagy.

### The NBV-MB nonstructural protein μNS contributes to autophagy induction

**Fig. 4:**
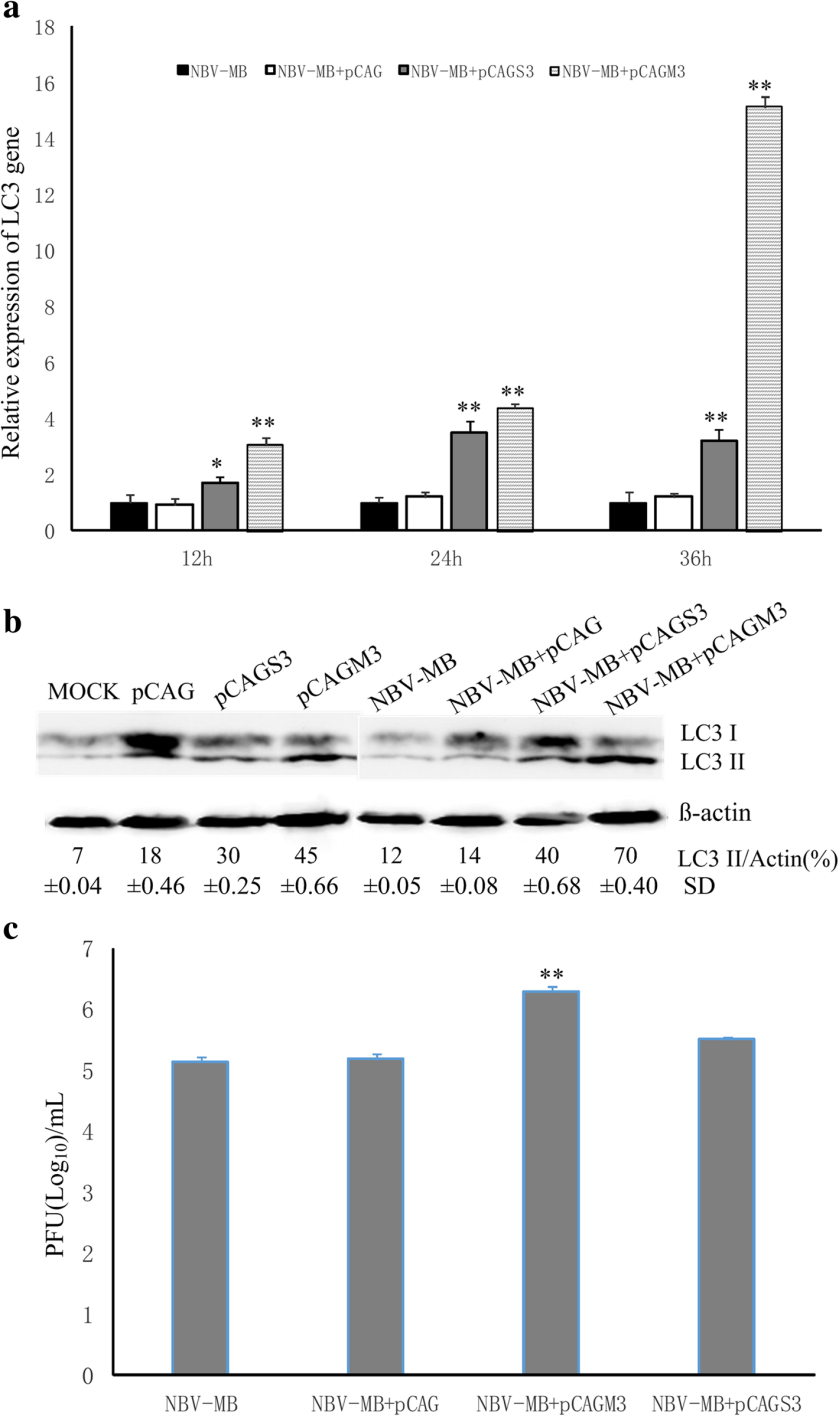
The nonstructural protein μNS contributes to induction of autophagy by NBV-MB infection. BHK cells in 6-well plates were transfected with pCAGM3 or pCAGS3; after 24 h, the cells were infected with NBV-MB at a MOI of 1. After 24 h of infection, cellular RNA was extracted and subjected to real-time PCR to measure the level of *LC3* mRNA (**a**). Cell lysates obtained after 24 h of infection were analyzed by western blotting with specific antibodies against LC3-I/II and β-actin (**b**). Viral titer in supernatant was measured in a plaque assay (**c**). Similar results were obtained in three independent experiments

Duck reovirus σNS triggers autophagy and promotes virus replication [26]. In light of this observation, we investigated the roles of NBV-MB nonstructural proteins in autophagy. The two major nonstructural proteins used in this study, μNS and σNS, are encoded by M3 and S3, respectively. Accordingly, BHK cells were transfected with pCAGM3, pCAGS3, pCAG (negative control), or nothing (control). In parallel, other samples were infected with NBV-MB at a MOI of 1. At 24 h post-infection, cellular RNA was extracted for real-time PCR to measure expression of *LC3* mRNA, and cell lysates were collected for western blotting to measure expression of LC3-II protein. As shown in Fig. 4a, compared with cells infected with virus alone, expression of *LC3* mRNA was higher in cells transfected with pCAGM3 or pCAGS3 plasmid before infection, particularly in cells transfected with pCAGM3. Furthermore, expression of *LC3* mRNA increased over time, reaching a peak 36 h post-infection. Relative to mock-infected cells or cells infected with NBV-MB after transfected with pCAG, expression of LC3-II protein was also elevated in cells infected with NBV-MB after transfected with pCAGM3 or pCAGS3 (Fig. 4b). The viral titer in cells transfected with pCAGM3 before infection was significantly higher than that in cells transfected with pCAGS3 or in cells infected with virus alone. The titer in pCAGS3 plasmid-transfected cells was somewhat higher than that in virus-only cells, but the difference was not significant (Fig. 4c). Taken together, these results suggest that NBV-MB infection triggers autophagy. In addition, the nonstructural protein μNS of NBV-MB may augment autophagy during viral infection.

## Discussion

The NBV-MB isolated from a patient with acute respiratory infection, can cause lethal outcomes with severe pneumonia in adult immunocompetent inbred mice when administered intranasally but not orally, indicating that NBV might cause respiratory diseases [34]. Hence, it is possible that NBV might be transmitted among humans, resulting in disease. The strong pathogenic properties of NBV relative to other reoviruses may be related to the interaction between NBV infection and the host cell response. Autophagy, as a cellular adaptive response, is involved in various aspects of viral infections [35, 36], including restriction of the intracellular pathogen replication. Thus, autophagy is a newly recognized facet of innate and adaptive immunity against viral infection. Despite the importance of autophagy as a component of host defense, certain viruses have developed strategies to counteract these antiviral mechanisms, whereas others have co-opted the autophagy machinery as proviral host factors that promote viral replication. Autophagy has been examined in avian reovirus and MRV infection [24], but previously the relationship between NBV infection and autophagy had not been examined. Because the autophagic response to different strains of a virus is not always the same, the effect of NBV infection on autophagy should be studied further.

We observed that formation of autophagosome-like double-membrane vesicles and the conversion of LC3*-*I to LC3*-*II were activated in NBV-MB-infected cells. Moreover, NBV-MB -infected cells accumulated greater numbers of GFP-LC3 puncta. Many viruses can induce autophagy [24, 36–39], and some reap the benefits to facilitate their own replication [24, 25, 37, 38]. Wu et al. reported that Muscovy duck reovirus (MDRV) induces autophagy in DF-1 and MDEF cells, and that autophagy promoted virus production; the σNS protein plays a critical role in this effect [26]. In addition, as noted above, avian reovirus can induce autophagy in both Vero and primary CEFs. The class I phosphoinositide 3-kinase (PI3K)/Akt/mTOR pathway contributes to induction of autophagy by ARV infection [24]. Li et al. also showed that p17, the nonstructural protein encoded by the S1 gene of avian reovirus (ARV), induces autophagy in infected cells, and that this process increases the rate of viral replication [40]. The p53/PTEN/mTORC1, AMPK, and PKR/eIF2α signaling pathways are involved in autophagy induction by p17 [25]. In our study, we also found that μNS protein participated in induction of autophagy by NBV-MB. Inactivated virus could not promote autophagy, indicating that induction of autophagy depended on productive virus replication. Conversely, autophagy increased viral replication, as demonstrated by the effects of both treatment with autophagy regulators and transfection of μNS plasmids. μNS and the autophagy inducer RAPA increased viral replication, whereas the autophagy inhibitors CQ and 3-MA had a non-significant negative effect. The underlying reason for this remains unknown.

Autophagy promotes NBV-MB replication, but the signal pathways involved in this phenomenon remain to be elucidated. In contrast to NBV-MB infection, WNV infection induces autophagy, but this induction causes WNV replication to be inhibited at the genome replication and gene expression stages [10]. The mechanisms by which WNV replication is inhibited by autophagy are unknown [10]. In the cases of some viruses, such as vesicular stomatitis virus and chikungunya virus, the autophagic pathway can also play an antiviral role [16, 41]. Possible antiviral functions include digestion of intracytoplasmic viral components (virophagy) or activation of innate and adaptive immunity by presentation of viral molecules [22]. In addition, autophagy stimulates production of type I interferon (IFN) [42, 43], which inhibits WNV genome replication and gene expression [44].

NBV might be among the viruses such as poliovirus, dengue virus, and HCV that have evolved mechanisms to escape host autophagy and use components of the autophagic machinery for replication [45–47]. Several lines of evidence suggest that some RNA viruses can utilize or manipulate cellular processes to facilitate their own survival and replication [19, 48]. For some viruses, intracellular membranes might be needed as scaffolds for viral replication within the cytoplasm [49]. Autophagy can direct trafficking of vesicles to new locations within the cell, which helps the virus to replicate. In the case of hepatitis C virus (HCV), autophagy promotes both viral protein translation and viral replication [46, 50]. In addition to RNA viruses, DNA viruses can also use autophagy for replication. For example, adenovirus and the E7 protein of human papillomavirus 16 (HPV16) induce autophagy. Activation of autophagy may result in cell death, thereby promoting release of viral progeny [18, 51]. The mechanisms by which autophagy increases NBV replication and propagation remain unknown.

In summary, we have provided the first evidence that autophagy is induced by NBV-MB infection and that induction increases the replication of NBV-MB, suggesting that NBV uses the autophagic pathway to promote its own replication. Our findings are similar to observations reported previously for ARV and MRV. The autophagy inducer RAPA promoted NBV-MB replication, but autophagy inhibitors did not significantly decrease replication. The nonstructural protein μNS plays an important role in NBV-MB-induced autophagy. These data provide a foundation for future studies aimed at elucidating the molecular mechanisms of NBV-induced autophagy and the signaling pathways by which autophagy promotes NBV-MB replication.

## Conclusions

We showed that autophagy was induced in BHK cells upon infection by NBV-MB by measuring expression of LC3*-*II and GFP-LC3. The autophagy inducer RAPA increased the viral titer. By contrast, autophagy inhibitors 3-MA and CQ decreased the viral titer. In addition, the nonstructural protein μNS may contribute to autophagy induction.

## Data Availability

All data generated or analyzed during this study are included in the published article.

## Abbreviations

3-MA: 3-methyladenine
ARV: Avian orthoreovirus
BroV: Broome reovirus
BRV: Baboon orthoreovirus
CPE: Cytopathic effect
CQ: Chloroquine
CQ: Chloroquine
DMEM: Dulbecco’s modified Eagle’s medium
dsRNA: Double-stranded RNA
EBV: Epstein-Barr virus
FBS: Fetal bovine serum
HBV: Hepatitis B virus
HCV: Hepatitis C virus
HPV16: Human papillomavirus 16
HRP: Horseradish peroxidase
IFA: Immunofluorescence assay
KSHV: Kaposi’s sarcoma-associated human herpes virus type 8
LC3-II: Microtubule-associated protein 1 light chain 3 type II
MOI: Multiplicity of infection
MRV: Mammalian orthoreovirus
NBV: Nelson Bay virus
PFU: Plaque-forming unit
PKR: Protein kinase R
RAPA: Rapamycin
WNV: West Nile virus
